# The where of handovers by humans: Effect of partner characteristics, distance and visual feedback

**DOI:** 10.1371/journal.pone.0217129

**Published:** 2019-06-21

**Authors:** Saki Kato, Natsuki Yamanobe, Gentiane Venture, Eiichi Yoshida, Gowrishankar Ganesh

**Affiliations:** 1 Graduate School of Engineering, Mechanical Systems Engineering, Tokyo University of Agriculture and Technology, Tokyo, Japan; 2 CNRS-AIST JRL (Joint Robotics Laboratory), UMI3218/RL, Tsukuba, Japan; 3 Manipulation Research Group, Intelligent Systems Research Institute, National Institute of Advanced Industrial Science and Technology(AIST), Tsukuba, Japan; 4 CNRS-University of Montpellier, LIRMM Interactive Digital Human group, Montpellier, France; University of Electronic Science and Technology of China, CHINA

## Abstract

Object handovers between humans are common in our daily life but the mechanisms underlying handovers are still largely unclear. A good understanding of these mechanisms is important not only for a better understanding of human social behaviors, but also for the prospect of an automatized society in which machines will need to perform similar objects exchanges with humans. In this paper, we analyzed how humans determine the location of object transfer during handovers- to determine whether they can predict the preferred handover location of a partner, the variation of this prediction in 3D space, and to examine how much of a role vision plays in the whole process. For this we developed a paradigm that allows us to compare handovers by humans with and without on-line visual feedback. Our results show that humans have the surprising ability to modulate their handover location according to partners they have just met such that the resulting handover errors are in the order of few centimeters, even in the absence of vision. The handover errors are least along the axis joining the two partners, suggesting a limited role for visual feedback in this direction. Finally, we show that the handover locations are explained very well by a linear model considering the heights, genders and social dominances of the two partners, and the distance between them. We developed separate models for the behavior of ‘givers’ and ‘receivers’ and discuss how the behavior of the same individual changes depending on his role in the handover.

## Introduction

A handover is a complicated interaction between two agents in which one agent passes an object to another in time and space. Handovers are fundamental in human society and occur multiple times in our daily life. They are common in most service tasks, ranging from receiving money at a teller, passing and receiving of tools by an assistant, and serving of food by a caregiver [1]. However, object handovers between humans have been sparsely investigated, and the mechanisms underlying this fundamental human social task are still largely unclear. With the service industry being increasingly automated, handovers are also becoming an essential skill for robots. The key requirement of automated handovers is that they are perceived as comfortable and safe by their human partners, and an examination of handovers between humans can arguably help a lot in the regard [1].

A human handover starts from the time an intention to pass or receive an object is generated [2], which is followed by the hand movements both by the giver and the receiver to a particular position where the object transfer will take place. Finally, there is a physical interaction in which the object is transferred from one hand to the other [3]. Previous studies have examined issues regarding the velocity [4, 5] and trajectory [6, 7] of handover movements, and the grip force [3, 8] and grasp points [9, 10], and location [11, 12] and configuration [13, 14] of the exchanged object. Here we were interested in how the specific physical and social characteristics of a partner influences handovers. In this study we concentrated on the *where* of the handovers- we examined how humans determine the position of handovers, and whether and how the handover position depends on who the interacting partner is, and how far he is. These two issues are still not clear from human hand over studies [15]. To address these issue, we looked at the contribution of ‘feedforward’ control in handovers between humans.

Human movements are widely accepted to be developed utilizing a ‘plan’ or so-called ‘feedforward’, as well as feedback control [16, 17]. Feedback control refers to the adjustment of movements using sensory observations, while the feedforward refers to his/her movement developed (even in the absence of feedback) using models or estimates of an environment, object and/or individual that a human has. The understanding of the contribution of feedforward and feedback in handovers can not only help in understanding the implicit effects of experience, partner modelling and social structures on handovers, but can help us optimize the design of machines for the same task. For example, in [18], humans experiments helped the authors to realize the importance of *reaction* feedbacks during physical interactions, which they then implemented in their robot controller. Similarly, understanding of the human ability to iteratively learn novel dynamics from current feedbacks [19] was used to develop a novel bio-mimetic algorithm for compliant interaction with human [20, 21].

Previous studies have shown that handover positions are modulated by the distance between the partners [15]. Handovers require one individual’s hand to enter the *peri-personal space* of another individual, which is a space around an individual’s body that they are known to be protective of [22], [23]Ṡtudies of peri-personal space has shown that it is affected by an individual’s reach [24] [25] and hence we hypothesized that an individual’s size and arm length to affect handovers. Intuitively, from day to day experience, we hypothesized handovers to also be affected by the gender and social dominance of the individuals, factors that we felt that previous studies have not investigated sufficiently. In this study we therefore investigated how humans determine the handover position of objects, by focusing on the following questions:

1. Whether a human, as a giver or receiver, has a model of the partner behavior, such that he/she can a-priori estimate the handover location a partner would choose?
2. Is this estimate better in certain directions than others?
3. Is the contribution of feedforward and feedback to handover different in different directions, and between a giver and a receiver?
4. And finally, how these issues are affected by the gender, physical size and social dominance of the interacting humans?

To address these questions, we asked twenty *participants* of different body sizes and social dominances [26] to give (and receive) objects from the same three unknown *representative partner*s or *partners* standing at one of three inter-personal distances (IDs). Crucially, after looking at each other’s position in each trial, we blind folded both the participant and the partner before making the handover action (giving or receiving) so as to prevent any online visual correction [27, 28]. We recorded the hand movements made by the participant during the handover, examined for systematic changes in the participant behavior with each partner, and compared these behaviors with those when their eyes were open. To anticipate our results, we observed that humans can immediately estimate the handover position of partners they meet for the first time, both when they are a giver or a receiver. Interestingly, the estimation is better along the axis joining the two individuals, than in the planes perpendicular to it. A giver seems to rely more on feedback than a receiver. And finally, these behaviors are well explained by a linear model considering the heights, genders and social dominances of the interacting individuals, and the distance between them.

## Materials and methods

### Subjects

26 subjects participated in our experiment. The subjects were divided into two groups. Each group involved 13 individuals, 10 as *participants* and 3 as *representative partner*s or *partners* for short. All participants in a group interacted with every partner exchanging roles both as givers, and receivers. Note that, this procedure of all participants (in a group) interacting with the same three partners was chosen as apriori we did not known whether and which partner characteristics affect the handover, and this procedure enables us to use a 2 way ANOVA to see whether a particular partner is influencing the handover of the participants, without considering which characteristics of the partner. We took two groups with different partners to avoid fatigue in the partners who have to work with many participants without information about the purpose of the experiment.

Overall this procedure gave us data from thirty pairs in each group (and 60 in total), with the participants as givers, and as receivers respectively, and enabled us to make an intra-participant analysis to see whether and how their behaviors change with each partner. All sessions were carried out on the same day. Each session lasted about 25 minutes, such that the total experiment for a participant lasted 1.5 hrs.

The 10 participants in group 1 were all males (age of 23.7±1.3, height of 174±6.7 cm, arm length of 54.7±3.4 cm). They worked with 2 males and a female partner (partner1-male, 23 years, 171 cm height, 52 cm arm length; partner2-male, 25 years, 180 cm height, 58 cm arm length; partner3-female, 22 years, 168 cm height, 55 cm arm length). Group-2 included 4 males (age of 57.0±8.15, height of 169.3±5.12 cm, arm length of 53.0±1.73 cm) and 6 females (age of 42.2±17.1, height of 156.8±3.48 cm, arm length of 50.5±2.29 cm). They worked with 2 males and a female partner (details in S1 Table). All participants and partners were right handed and had normal or corrected to normal vision. They were recruited using social media and word of mouth. The participant’s ages (including the new batch) were between 20 and 72 years old and by profession were students, housewives, temporary workers and retirees. The representative partners were chosen so as to include both genders and covering a large range of heights. There was also the constraint of availability. We wanted each participant to work with all their three partners on the same day, which meant that we required all three representative partners to be available together on multiple days so as to work with the 10 participants in the group.

The experiments were approved by the local ethics committee at the National Institute of Advanced Industrial Science and Technology (AIST) in Tsukuba, Japan, and all participants read and signed an informed consent form along with the PLOS consent form for the usage of their images in the paper before taking part in the experiments. Participants were well instructed and informed with the experiment and task procedure. Both the participants as well as the partners were naive to the motive of the experiment.

### Apparatus

**Fig 1:**
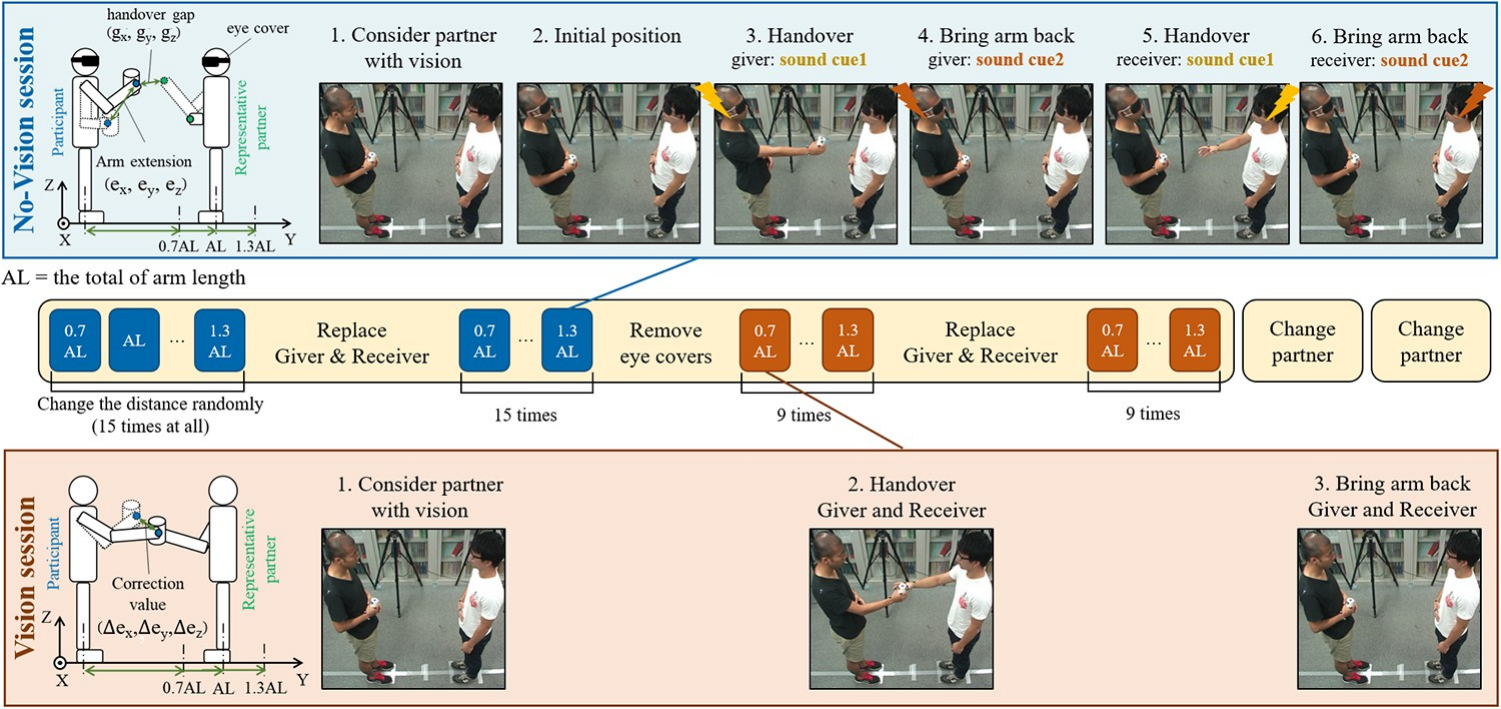
Experiment sequence. The top blue panel explains the No-Vision session (nVF), while the bottom red panel shows the Vision session (VF). The experimental outline and measured values are shown on the schema on the left. The experiment flow is explained in the photos on the right. And the central part explains the whole experimental sequence.

Fig 1 shows our experiment setup. Our experiments required the participants to either give an object to (as a giver), or receive an object from (as a receiver), a partner. A total of eight reflective markers of diameter (d = 15.0 mm) were fixed on the right arms of the participant and partner: One each on their shoulder, two each on their right wrists, and one on the metacarpophalangeal joint of each of their index fingers. A light (75.0 g), cylindrical, object with a diameter of 55 mm, height of 100 mm was used for the handovers. The giver was asked to utilize a “cylindrical grip” on the object during the handover. A reflective marker was attached to the top surface of the object and three markers were attached to the side of the object. A motion capture system with 6 cameras (Kestrel) recorded the handover tasks with a resolution 2048×1088 pixel, at 300 fps. We will concentrate on the hand movements, and hence the metacarpophalangeal joint marker (or MP marker) on the right index finger in this study (see details in S1 Fig).

### Experiment sessions

#### No-vision (nVF) sessions

**Fig 2:**
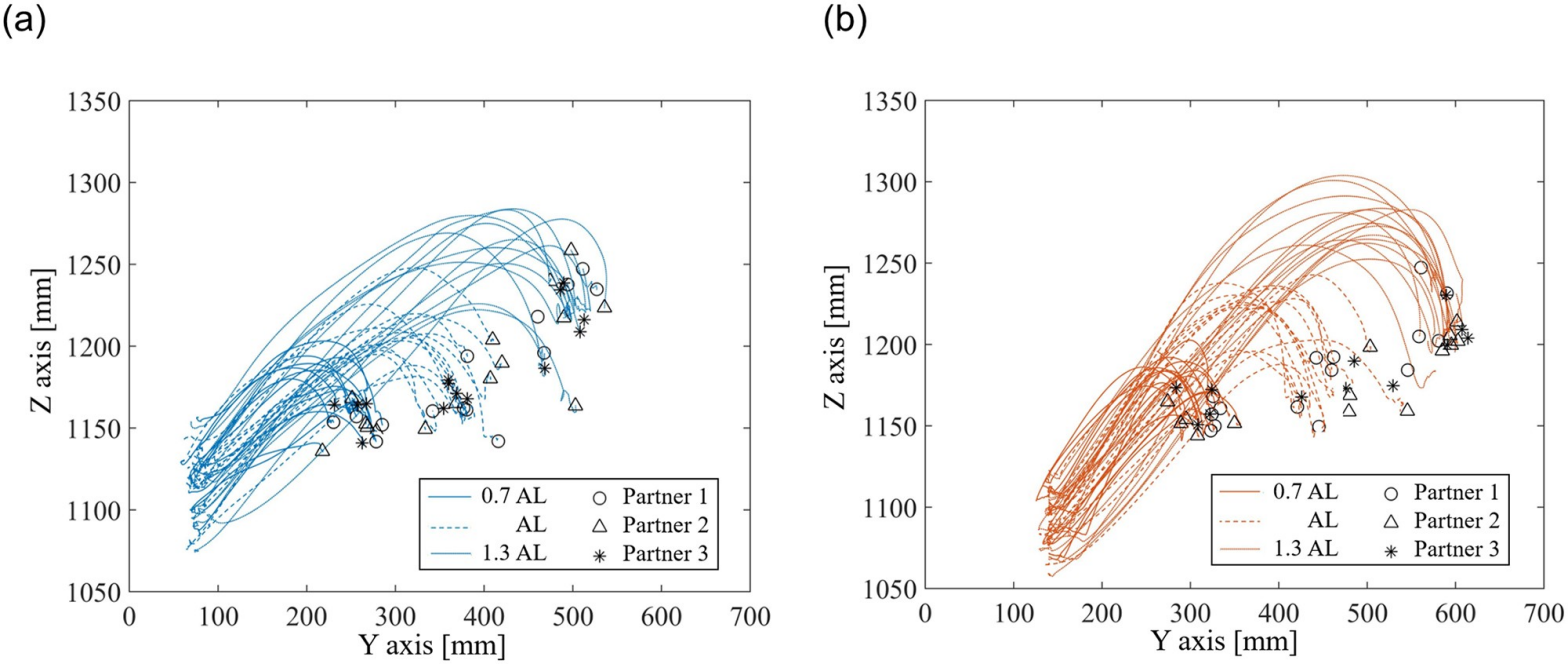
Handover trajectories in the no-vision sessions. The trajectories represent the movements of the marker on the right finger of a representative participant, recorded using the motion tracking system, when he worked as a (a) giver, and (b) a receiver. The different lines represent different inter-personal distances, and the different markers represent trajectories with different representative partners.

The experiment started with the measurement of the arm lengths of the partner and participant, which were used to define the distance AL (as in [15]). A mark on the floor indicated the position where the receiver should stand, while three marks were made for the giver at three inter-personal distances (or IDs) of 0.7AL, AL and 1.3 AL. In each handover, the receiver stood upright at the receiver mark on the floor. An experimenter orally announced the mark to which the giver was supposed to move. Both the receiver and the giver (with the object in hand) were then required to take their initial position, with their right hand touching their stomachs. They were asked to look at the other, and then cover their eyes with an eye cover provided to them. Both the participant and partner wore a wireless earbud in their ear (SoundPEATS Q29) and were instructed to “make your handover movement when you hear an audio tone, hold the extended arm (they held for about 4.0 seconds), and bring your arm back when you hear a second audio tone. Consider your partner and his position (from before putting on the eye cover), and make a smooth giving/receiving movement without thinking too much”. They were instructed that “you are required to keep your standing position unchanged during the handover, though you are allowed to move your hip and bend your back if you feel necessary”. Finally, they were explicitly told that “as your eyes are closed, it is normal that your hands do not make contact. Please do not worry about it as this is what we want to investigate, and hence, please do not try to make/improve contact across hand over trials”. Unknown to individuals, we introduced a delay between the cues to the two subjects, which ensured that the participant and partner made their handover movements one after another and their hands never collided. The object remained with the giver throughout this session and was never passed to the receiver. The experiment sequence is shown in Fig 1. The movements of a marker on a representative participant’s hand (explained later) during handovers over different IDs to different partners are shown in Fig 2a and 2b, when he acted as the giver and as a receiver respectively.

Following each handover, the individuals removed their eye cover and the experimenter announced the next mark for the receiver to move to, and start the next handover. The order of the receiver positions was selected in a pseudo-random order that the subjects could not predict.

#### Vision (VF) sessions

The procedure followed for the handover was similar in the vision sessions (see Fig 1) with the exception that the subjects did not cover their eyes and they made their respective hand movements naturally together, again cued by audio signals (which were no longer time delayed). Furthermore, in the VF sessions, the subjects were instructed to “extend your arms when you hear the audio cue, make sure you reach your partner’s hand, and then hold your movement at the point where both of you have taken hold of the object. On hearing the next cue, bring back your arm without giving/taking the object”. Therefore again, the object remained with the giver throughout the session.

#### Session order

Each participant in our study interacted with every partner over multiple sessions, both as a giver, and receiver, and with or without visual feedback. Furthermore, each session included an equal number of handovers made over three IDs- equivalent to 0.7AL, AL and 1.3 AL. Therefore, each participant worked in 3 sessions (with 3 partners)×2 (as giver and receiver)×2 (nVF and VF) = 12 sessions. The nVF sessions included 15 (5 × 3 IDs) handovers, and the VF sessions included 9 (3 × 3 IDs) handovers.

While each participant worked with all the three partners, the order of the partners they worked with, was randomized across the participants. The participants completed all the nVF and VF sessions with one partner, before working with the next. With each partner, participants performed the nVF sessions first, both as a giver and a receiver (the order was randomized across subjects). This was followed by the VF sessions in which the same order (of either giver or receiver first) was maintained.

#### Social dominance questionnaire

We measured the social dominance of the participants and partners using a Social Dominance Orientation questionnaire [26] (listed in). Social Dominance Orientation is a conceptualization of the attitudes of individuals among groups and was measured using a Likert scale (of 1-7) of 16 items. The participants and partners completed this questionnaire after the completion of the entire experiment.

### Data analysis and modeling

#### Observed variables

In this study, we report how the handovers by the participants changed when they were givers and receivers, with or without visual feedback, with respect to the gender, height and dominance of the participant and their partner. Specifically, we looked at three variables:

1. Arm extension (*e*): The arm extension defined how much the participant extended their arm in the nVF sessions during a handover, in the medio-lateral or X (*e*_*x*_), antero-posterior or Y (*e*_*y*_), and in-ferior-superior or Z (*e*_*z*_) directions. The arm extension was measured as the average difference between the position of the MP marker in the initial position and end of the hand over arm movement. The initial position of a participant or partner was defined as the position when the speed of the MP marker crossed over a threshold of 100 mm/s, and end position was defined as the position at which it fell below 100 mm/s for the first time after the start of the reach.
2. Handover gap (*g*): was defined as the absolute difference between the hand positions of the giver and receiver in the nVF sessions. It was measured as the average difference between the MP markers on a participant and partner at the end of every handover, in the X (*gap*_*x*_), Y (*gap*_*y*_), and Z (*gap*_*z*_) directions.
3. correction (Δ*e*): was defined as the difference between the participant’s hand at the end of their handover in the nVF session, and their hand position at the end of their handover in the VF session with the same partner, again in the X (Δ*e*_*x*_), Y (Δ*e*_*y*_), and Z (Δ*e*_*z*_) directions.

The giver sessions (where participants were givers, and the partners were receivers) and receiver sessions (in which the participants were receivers and the partners were givers) were analyzed independently across participants.

#### Analysis strategy

**Fig 3:**
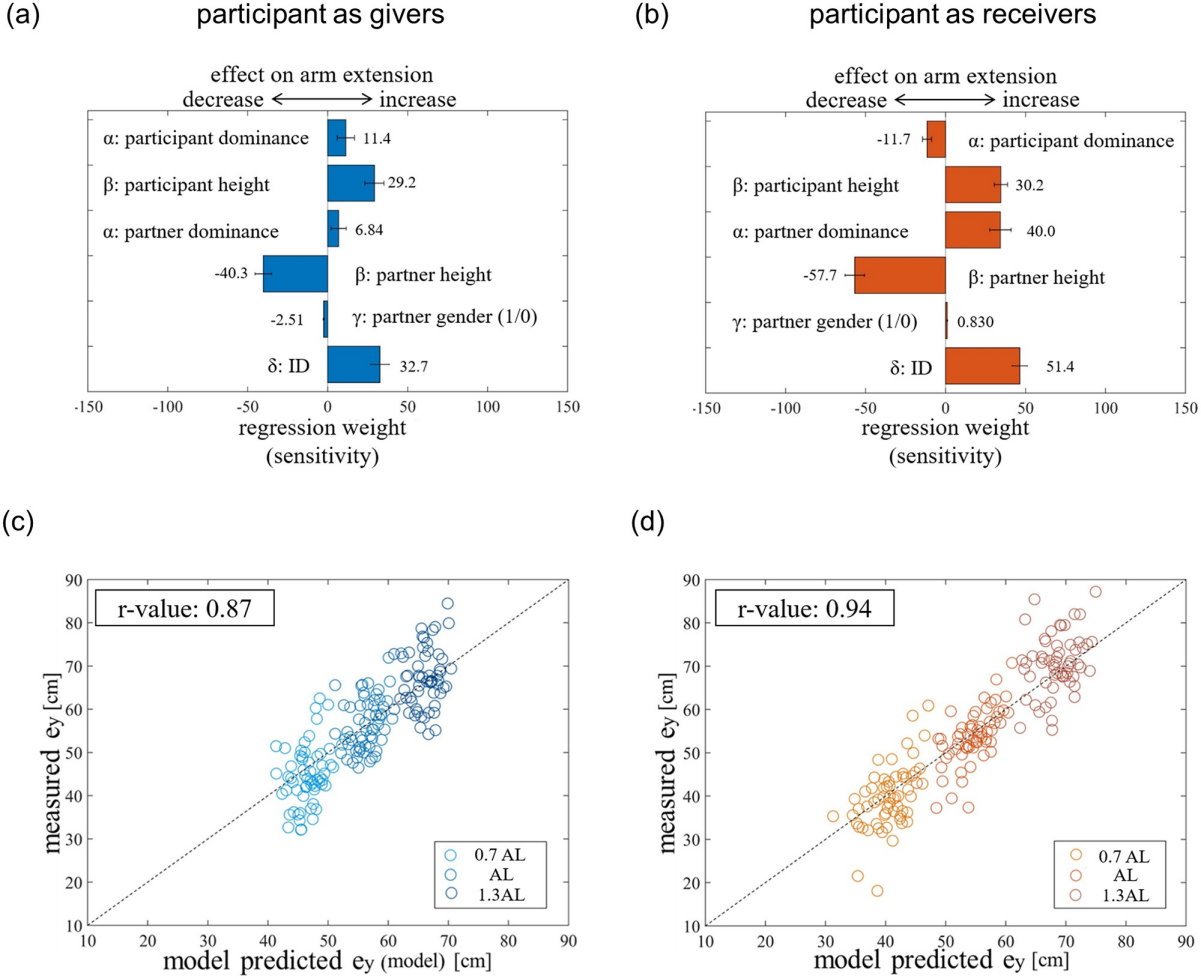
The model of arm extension e_y_. Separate multiple models were developed to explain the *e*_*y*_ by givers and receivers. The average and SE of the weight values of different variables in the linear models are shown for the (a) givers, and (b) receivers. A positive weight indicates that a higher value of the variable increases *e*_*y*_, while a negative weight indicates that a higher value of the variable leads to a decrease in *e*_*y*_. We examined the accuracy of our model’s prediction by plotting the model predicted *e*_*y*_ (with the average weight values), against the observed *e*_*y*_ by the (c) givers, and (d) receivers. The concentration of the data points on the 45 deg line indicates that the model is able to explain the data well.

**Fig 4:**
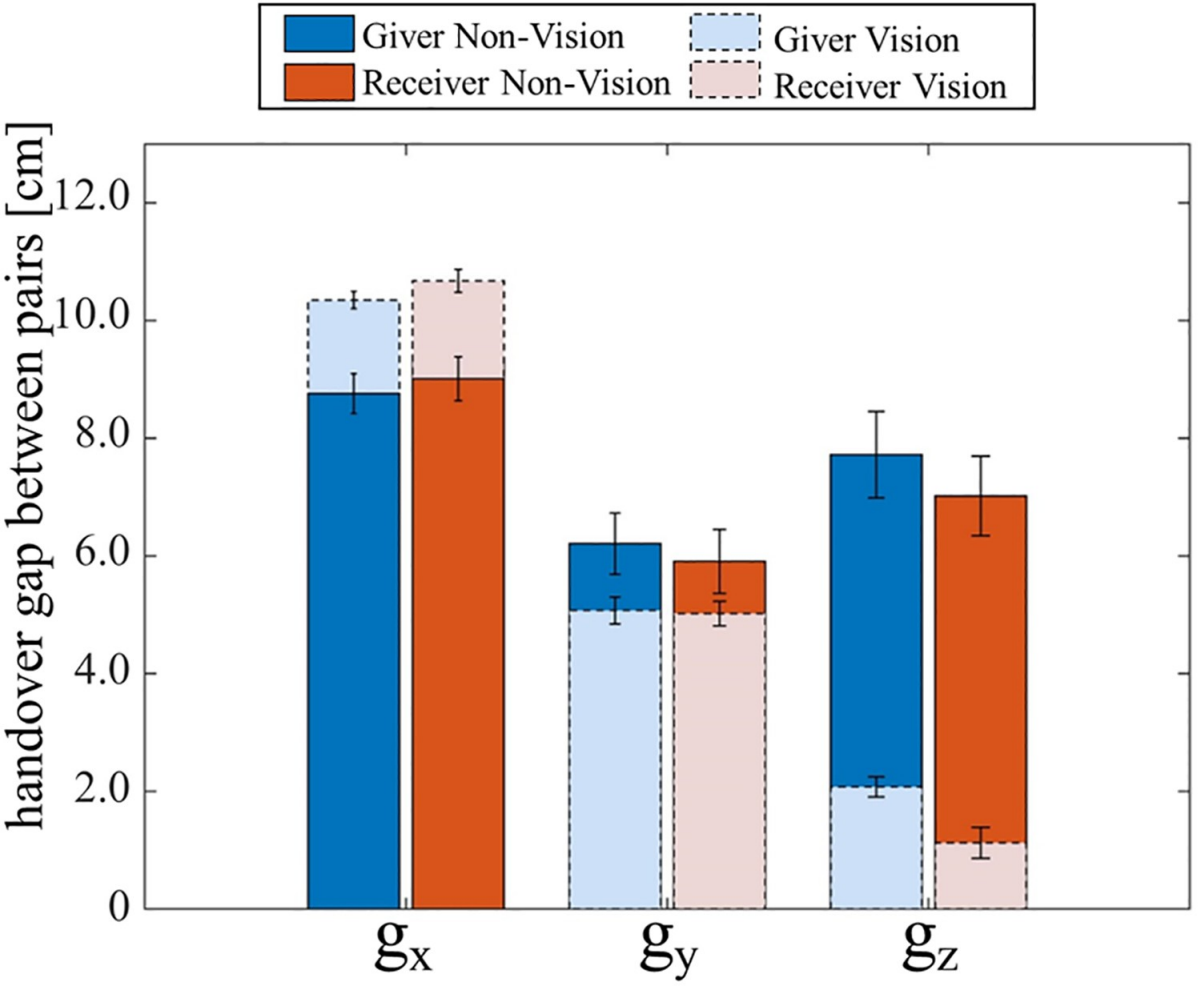
The handover gap remains relatively small and least in the Y direction. The dark shaded bars are nVF sessions, and the light shaded bars are the VF sessions. The handover gap in the y direction, *g*_*y*_, was consistently smaller than that in the X and Z directions. Note that the gap is non-zero in the VF sessions as it is measured between the MP markers. The fact that *g*_*y*_ is almost the same between the VF and nVF sessions shows that humans can predict the handover position in the Y axis even when nVF sessions.

**Fig 5:**
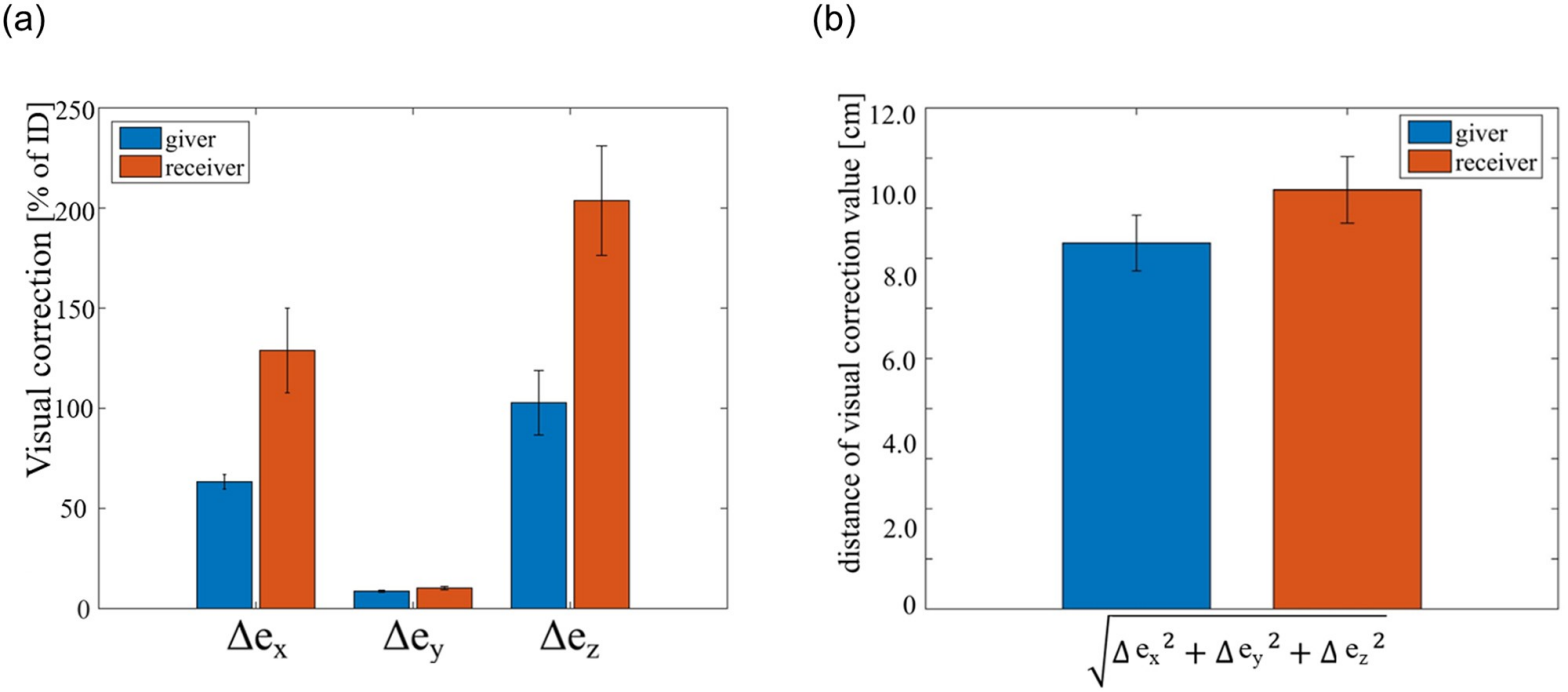
Visual correction: To estimate the contribution of visual feedback in hand overs, we looked at the ratio of the visual corrections by individual participants with each partner, and their initial handover gap with the same partner. This is plotted in (a) for the sessions when the participants were givers (red bars) and when they were receivers (blue bars). The overall correction values are shown in (b).

To analyze the data, we started first with a 2-way ANOVA of the arm extensions on the factors ‘ID’and ‘partner’. As mentioned before, we had each participant in a group interact with the same three partners, so as to enable an ANOVA on the factor ‘partner’, without requiring a hypothesis on the partner characteristics affecting the handover. We performed the 2-way ANOVA for each group separately. The results are shown in Table 1. Note that except for the ANOVA, we combine the data from both groups for all the other analysis. The model parameters (explained below), handover gap (*g*) and correction (Δ*e*) across all the 20 participants were plotted as an across participant mean and SE in Figs 3, 4 and 5 respectively.

**Table 1:** 2-way ANOVA on hand extensions in the X, Y, Z.

ANOVA	Factors	DOF	Giver p-value		Receiver p-value	
			Group1 N = 10	Group2 N = 9 (10)	Group1 N = 10	Group2 N = 10
e_*x*_	distance	(2, 16)	0.32	0.23 (0.20)	**1.1**×**10**^−3^	**1.5**×**10**^−3^
	partner	(2, 16)	0.42	**0.04 (0.02)**	0.43	**1.2**×**10**^−3^
	distance×partner	(4, 32)	0.46	0.19 (0.29)	**0.02**	**6.1**×**10**^−6^
e_*y*_	distance	(2, 16)	**2.9**×**10**^−8^	**1.1**×**10**^−9^ (**3.7**×**10**^−11^)	**4.0**×**10**^−10^	**2.2**×**10**^−16^
	partner	(2, 16)	**0.03**	**0.04** (0.08)	**8.9**×**10**^−3^	**2.3**×**10**^−5^
	distance×partner	(4, 32)	0.73	0.16 (0.07)	0.66	0.31
e_*z*_	distance	(2, 16)	**5.5**×**10**^−6^	**1.0**×**10**^−3^ (**2.9**×**10**^−4^)	**1.3**×**10**^−4^	**4.2**×**10**^−12^
	partner	(2, 16)	0.10	0.16 (0.07)	0.17	**1.7**×**10**^−4^
	distance×partner	(4, 32)	0.22	0.28 (0.23)	0.63	**0.02**

We created three linear regression models using the first 10 participants to understand how the antero-posterior arm extension (*e*_*y*_) of participants was modulated by the height, gender and dominance of a participant and partner, and the interpersonal distance ID between them. The self-only model assumed that the participants arm extension was modulated only by his own features (height, gender and dominance), and the ID. The other-only model assumed that the participants arm extension was modulated by his partner’s features (height, gender and dominance), and the ID. Finally, the interaction model assumed that the participants arm extension was modulated by both his own, as well as his, partner’s features (height, gender and dominance), and the ID. As the number of parameters are different between the models, we utilized the Akaike Information Criteria (AIC) to evaluate which model best explained the data. From this analysis we found that the interaction model was the best to explain the arm extensions, both when the partici-pants were givers (*AIC*_*interaction*_ = 1198, *AIC*_*self*–*only*_ = 1206, *AIC*_*other*–*only*_ = 1210), as well as when the participants were receivers (*AIC*_*interaction*_ = 1160, *AIC*_*self*–*only*_ = 1192, *AIC*_*other*–*only*_ = 1176). The interaction models utilized to explain the arm extensions (*e*_*y*_) were of the form:

$$\begin{array}{c} e_{y}=\alpha^{pt}\cdot D^{pt}+\beta^{pt}\cdot H^{pt}+\alpha^{pr}\cdot D^{pr}+\alpha^{pr}\cdot H^{pr}+\gamma^{pr}\cdot G^{pr}+\delta \cdot ID+\varepsilon \end{array}$$ (1)

Where D indicates the dominance score, and H the height of the participant (superscripted with pt) and partner (superscripted with pr), respectively. G represents the gender difference variable that took a value of ‘0’ when there was no gender difference between the participant and partner, and ‘1’ otherwise. *α*, *β*, *γ*, *δ* and *ε* are the weights of appropriate units.

#### Parameter estimation and model testing

We considered every combination of 18 participants (of the 20 participants) as training participants and calculated model parameters by linear regression, which were then tested with the remaining two participants. This procedure was repeated for every combination of 18 participants (190 combinations in total), and separately for when the participants were givers and receivers. To access the robustness of the estimated parameters and perform statistics, we analyzed the model fits on the training participants, as well as the prediction of the arm extensions of the distinct test participants, both with the *r*-values.

## Results

### Arm extension modulated by ID and partner

The mean observed arm extensions by the participants was equal *e*_*x*_ = −1.74±0.437 SE cm (when the participant is giver), *e*_*x*_ = 1.63±0.421 SE cm (when the participant is receiver), *e*_*y*_ = 56.4± 0.879 SE cm (when the participant is giver), *e*_*y*_ = 54.5±1.07 SE cm (when the participant is receiver), and *e*_*z*_ = 9.76±0.480 SE cm (when the participant is giver), *e*_*z*_ = 9.29±0.521 SE cm (when the participant is receiver), across participants (see Fig 1 for coordinate definition) of the two groups. We started with a conservative analysis, and performed separate 2-way ANOVAs on the arm extensions along the *e*_*x*_, *e*_*y*_, and *e*_*z*_ for the two groups separately. The two factors were ‘partner’ (which had 3 levels), and ID (which had 3 levels). The results are given in Table 1. First, we observed a significant main effect of ID on the arm extensions in both groups. Participants clearly modulated their handover behavior in the 3D space to account for the interpersonal distance, both as a giver and a receiver. Interestingly, we also observed a significant main effect of partner on the arm extension towards the partner, that is, *e*_*y*_ in both groups. The participants modulated their anterio-posterior arm extension *e*_*y*_ systematically according to the partner they interacted with. Though there was also an observed interaction between ID and partner in the lateral arm extensions *e*_*x*_, and *e*_*z*_, here we will concentrate on the effects on *e*_*y*_ because of the fact that the magnitudes of *e*_*x*_ and *e*_*z*_ are relatively small compared to *e*_*y*_, along which we varied the ID. A post hoc T-test revealed that participants in both groups changed their *e*_*y*_ for each partner when the participant was a giver as well as when he was a receiver (one-sample T-test between every two partner was observed to be p<0.001 in both groups, Bonferroni corrected).

### Evaluating factors affecting arm extension

Knowing that both the ID and the partner influence *e*_*y*_, we next investigated what aspect of the partner’s features affects the handover behavior. Specifically, we analyzed how the height and social dominance of the participant, and/or the partner, and the gender difference between them contributed to changes observed in the giver and receiver *e*_*y*_. We utilized linear regression for this analysis. We had also collected the arm lengths of the participants and partners, but could not include it in this analysis as it was found to be correlated to their height.

As mentioned before, AIC values showed that a model considering both participant’s and partner’s height, dominance and gender, and the ID explained our data best (see Eq (1)). Like mentioned in the methods, the regression was performed 190 times with this model using every combination of 18 (out of 20) participants, from which we achieved an average R-squared value of 0.69±0.001 SE across participants (*p* < 4.06 × 10^−23^) when the participants were givers and 0.87±0.030 SE across participants (*p* < 7.11 × 10^−31^) when the participants were receivers. The predictability of each model was tested on the remaining 2 distinct test participants. The fits had a root mean error of 5.32±0.02 SE cm with an r-value of 0.83±0.0005 SE (*p* < 4.8 × 10^−36^) for givers, and root mean error of 4.54±0.01 SE cm with an r-value of 0.94±0.0079 SE (*p* < 3.44 × 10^−57^) for receivers.

The across training regression weights on each factor of Eq (1) is plotted in Fig 3a and 3b. The weights calculated for the giver and receiver sessions show interesting differences between the behaviors of the same participants in the two roles. The participants modulated their *e*_*y*_ with ID, both when they were givers and when they were receivers (see lowest bars in Fig 3a and 3b). This was also predicted by the ANOVA (Table 1). The behavior of participants was not affected by the gender difference when they were receiver or giver. The height of the partner affected them both as a giver and a receiver- participants extended their hand less to give to and receive from a taller partner (4th bar in Fig 3a and 3b). A higher partner dominance led to an increase of the arm extension, both when the participant were givers and receivers (3rd bars in Fig 3a and 3b). Interestingly, however, the participants own dominance led to an increase of (*e*_*y*_) as a giver (1st bar, Fig 3a), but an decrease of *e*_*y*_ as a receiver (1st bar, Fig 3b). Finally, taller participants consistently showed increased *e*_*y*_, both when they were givers and receivers (2nd bar in Fig 3a and 3b).

Fig 3c and 3d show the model estimated *e*_*y*_ using the average parameter values from the 190 parameter estimations, plotted against the experimental *e*_*y*_ values for all the 20 participants. As can be seen from the concentration of the data on the 45 deg line, overall, the model fitted the data very well. The average RMS fitting error was 5.29±0.11 cm and 4.52±0.10 cm for when the participants were givers, and when they were receivers, respectively.

### Handover gap minimal in y

Our ANOVA and regression analysis above show that the participants modulate their arm extensions in the anterio-posterior direction (*e*_*y*_) depending on the physical and social characteristics of their partner. But why is this modulation required and relevant? The obvious possibility is that the modulation occurs because the participants can estimate the anterio-posterior handover position of their partner, and as a consequence, adjust their hand position to meet their partner’s hand. If this hypothesis is true, and if the participants are able to estimate their partner’s hand movement well, then we expected the hand over gap in the antero-posterior direction (*e*_*y*_, see Fig 1), to be relatively small. This was indeed the case. *g*_*y*_ was observed to be in the order of 5 cm (see dark shade bars in Fig 4), even in the nVF conditions. Note that *g*_*y*_ is measured as the distance between the MP markers on the hands, which would never reach zero even when the hands of the participant and partner do make contact. The *g*_*y*_ was in fact observed to be equal to 4.46±0.30 cm in the *y* direction during the VF sessions when the handover was executed successfully (see light shaded bars in Fig 4). Therefore, *g*_*y*_ was observed to be effectively around just 1 cm across the participants in the nVF sessions. Effective *g*_*x*_ was similarly low across subjects (see first bars in Fig 4), while a relatively large effective error of around 9.22±0.34 cm was observed in the *g*_*z*_. These values were significantly more than the gap in the *y* direction (*T*(29) = 3.75, *p* < 0.001(X-Y), *T*(29) = −2.69, *p* = 0.012(Y-Z)). Overall the effective gap between the participant and partners through our experiment was 15.9±0.70SE cm.

Finally, we evaluated how the participant handover positions changed when they had vision (VF sessions), compared to when they did not (nVF sessions). Overall, across the participants, the visual corrections were equal to 2.82±1.65 STD cm in the *x* direction (Δ*e*_*x*_), 4.95±3.14 STD cm in the *y* direction (Δ*e*_*y*_), and 4.20±0.205 STD cm in the *z* direction (Δ*e*_*z*_) respectively. In Fig 5A, we present Δ*e*_*x*_, Δ*e*_*y*_, Δ*e*_*z*_ as ratios, by dividing the visual correction value by the corresponding handover gap (*e*_*x*_, *e*_*y*_ *and* *e*_*z*_). It is interesting to note that, in the *y* direction, given that the distance between the participant and partner was on average equal to one ID, the contribution of the feedback correction was only 4.25±0.32 STD% of the ID. On the other hand, visual *z* corrections Δ*e*_*z*_ were almost 103 % (when the participant is giver) /204 % (when the participant is receiver) of the initial *e*_*z*_, and Δ*e*_*x*_ was around 63 % (when the participant is giver) /129 % (when the participant is receiver) of the initial *e*_*x*_. As *e*_*x*_ was not modulated in this experiment and was a-priori small, we cannot say much about the contribution of feedback to Δ*e*_*x*_. But our results show that during the handovers, the human arm movements in z, *e*_*z*_, largely relies on the visual feedback, while the visual feedback does not contribute much for *e*_*y*_.

Across participants, we observed that the visual corrections made by the same participants as a receiver, were significantly more (*T*(19) = −1.65, *p* = 0.10) than what they made as a giver (Fig 5B), though practically the differences were in the order of few centimeters.

## Discussion

In our study we modulated the partners and the handover distances for participants, and analyzed how this affected their hand over position as a giver or as a receiver. Specifically, if participants are able to a-priori estimate their partner’s preferred hand over position. To avoid contamination due to visual feedback, which is arguably a major contribution in the handovers, we adopted a strategy utilized in many motor control studies, that to evaluate the participant handovers in the absence of visual feedback.

We observed that the handover positions can be explained surprisingly well by a linear function considering the distance from their partner, and the participant’s, as well as their partner’s, physical and social characteristics (Table 1, Fig 3). While previous studies have proposed models for describing the handover position considering the physical characteristics of the participant [11] as well the inter-personal distance [15], our study is probably the first to show the influence of partner characteristics, especially social dominance, in human handovers (see third bar in Fig 3a and 3b). This result suggests that human participants can immediately estimate the preferred handover positions of partners they have just met. The fact that one’s behavior is influenced by the estimation of behaviors observed in others is well known. Such observations have been previously reported in society [26] and sports [29, 30], and during physical motor interactions [4]. It is therefore not surprising that we find the presence of a similar ability during handovers, which is arguably both a social and motor task. However, the fact that the participants were able to estimate the physical and social dominance of their partners so quickly, without much interaction with them, is surprising.

The participant’s ability to estimate the partner’s social dominance is especially intriguing because we measured the social dominance using a Social Dominance Orientation questionnaire [26]. The questionnaire assesses social dominance by asking individuals to rate their behavior in various social circumstances(S3 Table). The participant and partners do not have a chance to see each other’s rating, and do not have a chance to speak much to their partner as our protocol required. However, our modeling suggests that they are still able to estimate the other’s social dominance, probably by the observation of their partner’s behavior when they were given the experiment instructions. Furthermore, it is to be noted that the social dominance we measured (and used in the model) is the *self-perceived* dominance of the individuals, which can be different from the third person (participant) perceived dominance. Our study thus suggests the either, the first person and third person perceived dominance do not differ much (which though is unlikely), or that the participants are able to estimate the self-perceived dominance of their partners. However, further studies are required to clarify this issue.

In our experiment, we recruited a wide range of participants in terms of age and background. The fact that we can still explain their behavior well with one parameter set may be seen as showing the robustness of ID, and height and dominance of the participant and partner. On the other hand, the remaining fitting error suggests the presence of factors that we still miss in the model.

Finally, we also tried to estimate the contribution of visual feedback in handovers in the transport phase of the handovers. For this, we compared the handovers made by individuals with visual feedback and without visual feedback (Fig 5A), in which case the movement are feedforward. This procedure of course assumes that the feedforward is additive to the feedback. This is probably not the case in practice, where the hand movement control probably changes completely in the presence of vision. Still, Fig 5A does give us an estimate of the importance of visual feedback, and hence the partner behavior estimation, in the different directions. This information is crucial for the design of handovers by robots. For example, our results show that it is sufficient for robots to control the medio-lateral (*e*_*x*_) and inferior-superior or (*e*_*z*_) movements during handovers by visual servoing, which is relatively easy for robots, as humans seem to do the same. On the other hand, when it comes to the anterio-posterior movement (*e*_*y*_), robots need to have a good understanding of the human behav-ior, because the human’s behavior in this direction may be influenced heavily by the movement estimation and perception of the robot partner.

## Supporting information

S1 Table The information of participants and representative partners. (TIFF)

S2 Table Social Dominance Orientation items on the 16-Item Social Dominance Orientation Scale [26]. (TIFF)

S3 Table Details of the SDO questionnaire result of the subjects. (TIFF)

S1 Fig The markers were placed on the radial styloid process and ulnar styloid process of the wrist. (TIFF)
