# Supplementary figures and images for: The where of handovers by humans: Effect of partner characteristics, distance and visual feedback
Source: PLoS One. 2019 Jun 21;14(6):e0217129. doi: 10.1371/journal.pone.0217129 (PMC6588208; doi:10.1371/journal.pone.0217129)

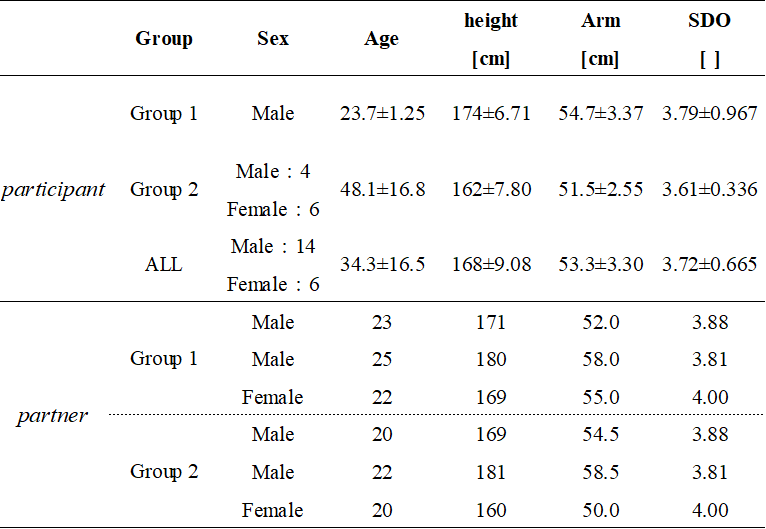

Supplement: S1 Table — (TIFF) [file pone.0217129.s001.tiff]

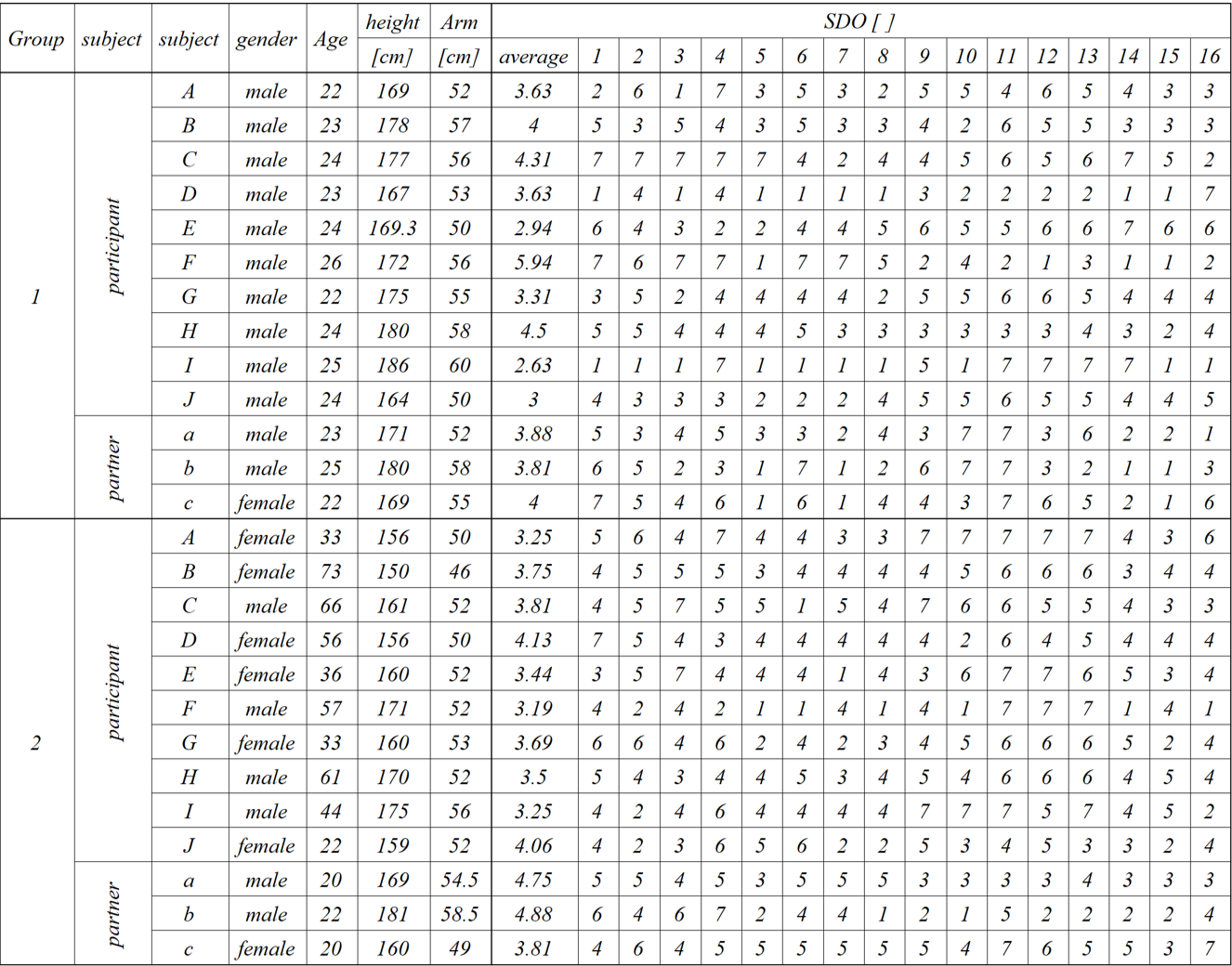

Supplement: S3 Table — (TIFF) [file pone.0217129.s003.tiff]

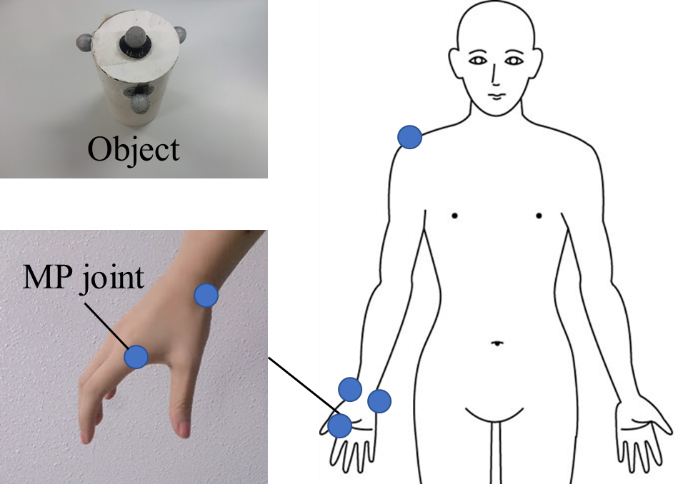

Supplement: S1 Fig — (TIFF) [file pone.0217129.s004.tiff]
